# Well-Being, Self-Esteem and Temporal Perspective in Incels: An Italian Study

**DOI:** 10.3390/jcm13020358

**Published:** 2024-01-09

**Authors:** Costanza Scaffidi Abbate, Federica Rapacciuolo, Silvana Miceli

**Affiliations:** Department of Psychology, Educational Science and Human Movement, University of Palermo, 90128 Palermo, Italy; federica.rapacciuolo@community.unipa.it (F.R.); silvana.miceli56@unipa.it (S.M.)

**Keywords:** incels, online forum, public health, self-esteem, temporal perspective, violent behavior, well-being

## Abstract

The global scholarly attention has shifted toward the phenomenon of inceldom (involuntary celibacy) due to violent incidents involving self-identified incels. There is a growing number of platforms promoting the proliferation of these ideologies, and cases of violence are becoming increasingly severe. This research constitutes one of the limited empirical investigations within an Italian context. This study aims to examine the mental well-being and its associations with self-esteem and temporal perspectives among individuals identifying as incels. Fifty-eighth male subjects aged between 18 and 45 years old participated in the study. Participants, recruited through online communication channels, completed three questionnaires focused on assessing mental well-being, self-esteem, and temporal orientation. The results reveal that incel subjects exhibit low self-esteem and are inclined toward a hedonistic present-focused perspective aimed at immediate gratification rather than future planning. Of note are the data related to the future temporal perspective, which does not show any predictive value on the well-being of incel subjects. Their ability to plan for the long term, defer immediate gratification, and control behavior through the anticipation and evaluation of possible consequences appears diminished. This study discusses the implications of developing targeted intervention programs, given that the incel phenomenon is becoming increasingly widespread. It is, therefore, crucial not to underestimate the potential threat that inceldom could pose in the future.

## 1. Introduction

In 2014, in California, Elliot Rodger killed six people and injured fourteen others before taking his own life. In his manifesto, he mentioned a ‘Day of Retribution’, during which he planned to eliminate those he envied the most [1]. On 12 August 2021, Jake Davison, a UK resident, opened fire on a crowd, resulting in the deaths of two women, two men, and a three-year-old girl, with two others injured, before taking his own life. Investigations revealed that Davison had posted several videos on YouTube expressing feelings of failure, despair, and life defeat, attributing his social isolation to women. Furthermore, on 23 April 2023, Alek Minassian, a 25-year-old of Armenian descent in Canada, drove a van into a crowd in Toronto, resulting in the deaths of 10 people and injuring 16 others. A computer science student at Seneca College, Minassian, was arrested minutes after the attack began, despite the brief timeframe that did not prevent the tragedy. His diary revealed deep suffering, signs of rejection by women, envy toward peers successful in romantic relationships, and a perception of violence as the only solution to his torments. These are just three cases of homicides committed by young individuals who identified with the incel ideology.

‘Incel’ is an abbreviation for “involuntary celibate,” referring to a member of an online subculture mostly composed of heterosexual men who identify as involuntary celibate, unable to have romantic or sexual relationships not by choice but due to various reasons, often attributed to external factors or society. These individuals presuppose a natural entitlement to sex, attributing the unavailability of a romantic or sexual partner to not being attractive according to specific criteria independent of their will [2]. The incel community operates predominantly online, providing an outlet for expressing misogynistic hostility, frustration, and blaming society for a perceived failure to include them [3]. Various media and researchers have criticized online incel forums for being misogynistic, racist, encouraging violence, spreading extremist views, and radicalizing their members [4]. The Southern Poverty Law Center has even described these internet sites as “part of the online male supremacist ecosystem,” and they are included in their list of hate groups [5].

The misogyny characterizing incel culture and its potential for violence have received extensive attention from the academic community [6,7], to the extent that a significant portion of scientific research has primarily focused on analyzing misogyny, often resorting to linguistic analysis in online contexts [8,9].

However, empirical research analyzing the psychological profile of incels is rather scarce. Recently, Stijelja and Mishara [10] conducted a review of studies that primarily investigated the psychological profile of incels, highlighting that until 2014, there were very few academic publications empirically analyzing crucial psychosocial variables and socio-behavioral patterns of incels [2,11]. The review begins with Donnelly et al. [12], which represents the first study in the scientific landscape to use the label “incel”. In this investigation, the main psychological characteristics outlining the profiles of incels involve feelings of insecurity, a lack of social skills, resignation, and depression. Donnelly et al. [12] adopted a lifespan perspective, using the concept of “sexual time sentiment” to characterize those who do not choose to be celibate but remain so. Several participants felt they had missed important life transitions, with passing years exacerbating their sense of being different from their peers. Other studies [3,13,14] have identified mental health issues, primarily untreated, such as depressive symptoms, autistic symptoms, post-traumatic stress, anxiety symptoms, and even suicidal ideation. The topics examined range from online misogynistic language [8,15] to the Big Five personality traits of incels [16] and their use of pornography [17]. Other studies have focused on incels’ experiences, recriminations, ideology, and the prevalence of mental health diagnoses [3,13]. Daly and Laskovtsov [18] conducted a series of in-depth qualitative interviews with some incels. Participants spoke about perceiving challenges to masculinity, feeling marginalized, or being treated as ‘subhuman’ due to their appearance and subsequently experiencing negative emotions associated with their incel identity, thereby influencing their online misogynistic hostility.

The term incel is a neologism coined in 1997 by a young student at Carleton University in Canada, who used it on one of her sites created to welcome individuals who shared difficulties in establishing sexual relationships. The “Alana’s Involuntary Celibacy Project” was initially conceived as a self-help group and intended for men and women who had difficulty finding sexual partners [19]. Initially, the incel condition had no gender connotation, it did not lead to any violence, and the platform was aimed at mutual support and the elimination of social stigmas such as virginity. Slowly, celibates generated their own culture by coming together and building communities on social platforms, mainly Reddit and 4Chan [20]. It is essential to point out that the community of involuntary celibates is a subculture, a branch of a large tree, the so-called “Manosphere,” whose fulcrum is the support of the rights of the male gender [21]. Not infrequently in this culture, it leads to anti-feminist and sexist visions, in which feminism is seen as a burden that crushes the male counterpart, and women are dehumanized and mortified [8,22,23,24,25].

A prominent feature of inceldom is the pill theory, which takes its name from a scene from the 1999 film The Matrix. In a scene from the film, the protagonist Neo must choose between the blue pill, with which he will wake up in his bed as if nothing had happened, therefore returning to a comfortable but fictitious life, or ingesting the red pill, which represents the world of reality and truth [26]. Incels who join the red pill believe they know the sad truth about what women want from a man: money, good looks, and a good reputation. Faced with this awareness, the incel individual has two different options: the first is to accept the sad truth that women enjoy greater privileges than men and that the world favors so-called alpha men rather than beta men. The second option leads to the “Black pill”, a nihilistic and extreme position according to which there is no possibility of success in conquering a woman, neither on a romantic nor sexual level, and consequently, there is complete resignation to the incel condition [27].

What characterizes communication within the forum is the use of terms or acronyms of a common language that strengthens the ingroup and chants against those perceived by the community as adversaries: women and “alpha” men. As regards the first category, jargon is manifested, leading to the mortification and dehumanization of the female gender [28]. Women can be defined with some extremely derogatory terms such as cum dumpsters, roasties, holes, bitches, whores, sluts, foid, short for female humanoid, and further insults that denote an extremely low consideration of women, like an object [8,29,30,31]. The “alphas”, exemplified by an internet meme called Chad [32], stereotypically represent good-looking, attractive, and pleasant men with women. Chad is seen as the rival who prevents incels (beta men) from conquering their female counterparts and therefore forces them to remain celibate.

## 2. Mental Well-Being and Self-Esteem

Well-being is a multidimensional construct integrating hedonic and eudaimonic aspects, supported by a network of positive emotions, relationships, autonomy, competence, purpose, and resilience [33]. The hedonic approach defines well-being as pleasure and avoiding pain [34]. The eudaimonic approach instead refers to the meaning and personal self-realization, understood as the actualization of one’s potential [33,35,36]. Both of these aspects contribute to the development of positive mental well-being, including affective, cognitive, and good psychological functioning aspects [37]. The research precisely considers the construct of mental well-being, a state enabling individuals to realize their abilities, cope with everyday life stressors, and work productively and profitably, contributing to their community. It encompasses several salient aspects: happiness as a subjective experience of high levels of positive affect and low levels of negative affect, life satisfaction [38], psychological well-functioning, self-realization, understanding as the actualization of one’s potential and pursuit of intrinsic goals such as autonomy, personal growth, and positive social relationships [37]. The underlying mechanisms of mental well-being draw from various psychological theories. Positive emotions, positive relationships, autonomy, competence, purpose, and resilience are integral components contributing to an individual’s overall sense of well-being [39].

The construct of self-esteem, intimately intertwined with the theme of well-being, is pivotal in shaping our mental and emotional health. Self-esteem, defined as the subjective assessment of one’s worth and capabilities, profoundly influences daily functioning, directing individuals toward varying states of well-being [40]. This evaluative judgment of oneself becomes a crucial determinant in navigating life’s challenges and opportunities. 

The associations between well-being and self-esteem are well documented in the current literature. Notably, the connection between self-esteem and mental health is particularly emphasized in the works of Orth and Robins [40], who explore the longitudinal implications of self-esteem on the development of anxiety and depression. Moreover, the review by Alsarrani et al. [41] emphasizes the positive impact of high self-esteem on subjective well-being, highlighting the role of self-esteem in fostering positive affect and life satisfaction. Complementary findings from a meta-analysis by [42] reinforce this association, indicating that interventions aimed at improving self-esteem positively affect overall well-being. In the realm of positive psychology, Diener [43] has explored the connection between self-esteem and flourishing, positing that individuals with higher self-esteem are more likely to experience a sense of fulfillment and purpose in their lives. Similarly, a study by Tkach and Lyubomirsky [44] contributes to understanding the relationship by revealing that enhancing self-esteem is linked to increased well-being and happiness. These examples underscore a consistent pattern in the literature, highlighting the bidirectional influences between well-being and self-esteem. The findings collectively suggest that fostering positive self-esteem can serve as a valuable avenue for enhancing overall well-being and mental health.

This research aims to analyze well-being, self-esteem, and the temporal perspective in individuals identifying as incels. Our motivation for emphasizing these variables stems from various compelling factors. Regarding well-being, a comprehensive review of Stijelja’s literature [2] revealed limited scientific research, apart from Speckhard and Ellenberg’s [14] and Daly and Laskovtsov’s [18] studies examining the potential for self-harm and suicide tendencies in incel posts, despite romantic relationships being a strong predictor of well-being [45]. Although research on incels is still in its infancy, some information about incels’ well-being can be inferred from results indicating that romantic loneliness is associated with lower well-being and negative emotions [46], and romantic loneliness is higher among individuals who perceive themselves as involuntarily single rather than voluntarily single [47]. 

Regarding self-esteem, as previously indicated, studies have identified self-esteem-seeking as a recurring theme and online forum participation as a source of self-esteem [9]. Additionally, typical features of incels—difficulty in interpersonal relationships, lack of self-confidence, fear of judgment, self-destructive behaviors, and difficulty in asserting themselves [10,14,18,48,49,50,51]—may conceal a more global and severe general problem in their self-assessment. Several studies have found that themes of loneliness, despair, and depression pervade incel forums [52,53], with many members openly discussing suicidal plans online [6,7,8,18,20,24,54,55,56]. These themes are closely related to negative self-assessments and signs of poor mental well-being.

Lastly, examining the temporal perspective of incels can help understand how their past and present experiences influence their expectations for the future. This could have significant implications for their motivation and general well-being. Specifically, the current research investigates the temporal perspective according to Zimbardo’s model [57]. This analysis assumes a crucial role because the adopted temporal perspective can determine different decision-making styles with different evolutionary and social outcomes, influencing the adoption of healthy lifestyles or, conversely, implying the enactment of high-risk health behaviors [58,59]. Empirical studies show, for instance, that individuals with a temporal perspective essentially focused on hedonistic present (PH) use, less protection for safe sexuality [60], are more involved in traffic accidents [38], and engage more in alcohol and drug use [58]. In general, subjects with high scores in the present hedonist (PH) dimension appear more motivated to achieve short-term goals compared to medium and long-term goals; they are more focused on immediate emotional gratification; and they experience difficulty controlling impulses, risking the development of various types of dependencies.

Conversely, subjects with a present fatalist (PF) temporal perspective believe they cannot change the present and passively let themselves be guided by events they perceive as inevitable [61,62]. Those with a future-oriented temporal perspective (F), on the other hand, tend to implement more health-protective behaviors due to their ability to defer immediate gratification and control behavior by anticipating and evaluating its possible consequences. The ability to think about the future is associated with multiple adaptive behaviors and simultaneously shows a negative correlation with engaging in high-risk behaviors [57]. The temporal perspective could influence how incels handle social relationships and self-esteem problems.

In summary, self-esteem, mental well-being, and the temporal perspective play a crucial role in how incels perceive not only themselves but also the surrounding world, and understanding their possible nuances can contribute to the development of targeted prevention and intervention programs.

## 3. Research Objective

The research aims to analyze well-being, self-esteem, and the temporal perspective in individuals identifying as incels. Moreover, it explores whether self-esteem and the temporal perspective serve as predictors of mental well-being. The specific hypotheses driving the research are as follows:

1. Incel individuals exhibit a low level of mental well-being. In comparison to the standardized Italian sample, incel individuals report lower levels of well-being, as measured by standardized well-being scales.
2. Incel individuals have low self-esteem. In comparison to the standardized Italian sample, incels exhibit lower scores on validated self-esteem measures, suggesting a diminished subjective assessment of their worth and capabilities.
3. Incels predominantly exhibit a present hedonistic temporal perspective and are less oriented toward the future: In comparison to the standardized Italian sample, incels score higher on measures assessing present hedonistic temporal perspectives, emphasizing immediate pleasure and gratification over long-term goals. The scores on future-oriented temporal perspective measures are significantly lower in incels, indicating a diminished focus on long-term consequences and planning.
4. Both self-esteem and the temporal perspective adopted by incel individuals are valid predictors of mental well-being.

## 4. Materials and Methods

### 4.1. Participants and Procedure 

Fifty-eighth male subjects, aged between 18 and 45 (M = 26.1; SD = 6.27) years participated in a multi-method research design. The educational level was notably high, with all participants having over 13 years of schooling, 38% of participants possessed a bachelor’s degree, while 15% held a master’s degree. The study was conducted using an online questionnaire, with participants recruited through the online platform “https://ilforumdeibrutti.forumfree.it (accessed on 10 January 2020)”. Informed consent was obtained, outlining the survey’s purpose and emphasizing participant anonymity. Participants were informed of their option to abstain from answering specific questions and to withdraw from survey participation at any point. Before completing the questionnaires, demographic information regarding age range and educational level was collected. The criteria for inclusion encompassed individuals who self-identified as incels. Given the exploratory nature of the research and the relatively limited literature on this population, inclusivity was prioritized to ensure a comprehensive representation of diverse experiences within the incel community. There were no stringent exclusion criteria to facilitate the broad inclusion of perspectives. In particular, the inclusion criteria for participation in the study were as follows: participants had to be active members of the online incel group. They must be at least 18 years old. Participants did not receive any remuneration for participating in the study. 

The determination of the sample size in the study followed a pragmatic approach, given the unique nature of the study population. Due to the inherent challenges associated with accessing and engaging with this specific group, a non-probability sampling method was employed. Consequently, the sample size was not pre-determined through a formal calculation method, but rather, an exhaustive effort was made to include as many willing and eligible participants as possible. Thus, the sample construction in this study involved convenience sampling. Utilizing convenience sampling allowed us to reach potential participants more effectively, considering the difficulties associated with identifying and engaging individuals within this community. While this method acknowledges its inherent limitations, it was chosen strategically to maximize participation and obtain a diverse representation of incel perspectives.

### 4.2. Measure

Mental well-being was measured using the Warwick–Edinburgh Mental Well-Being Scale (WEMWBS) [37], adapted for use in Italy by Gremigni and Stewart-Brown [63]. The WEMWBS measures an individual’s mental well-being across various dimensions. Comprising 14 items in its original version, it assesses positive affect, interpersonal relationships, and a sense of purpose (i.e., “I’ve been feeling optimistic about the future”). Participants rate their experiences on a 5-point Likert scale. The WEMWBS provides valuable insights into mental health and is adaptable for cross-cultural use. The Italian version comprises 12 items [64]. The Cronbach’s alphas was 0.86.

Self-esteem was measured using the Rosenberg Self-Esteem Scale [64], adapted for use in Italy by Prezza et al. [65]. The Rosenberg Self-Esteem Scale is a widely utilized psychological tool designed to assess an individual’s overall self-worth and confidence; it consists of 10 items, each measured on a 4-point Likert scale (i.e., “On the whole, I am satisfied with myself”). The scale explores feelings of self-respect, acceptance, and a positive self-image. The Cronbach’s alpha was 0.91.

The Stanford Time Perspective Inventory by Zimbardo and Boyd [38], adapted for use in Italy by D’Alessio et al. [66], comprises 22 items with responses rated on a 5-point Likert scale. The scale assesses individuals’ temporal orientations, measuring their past, present, and future time perspectives to understand how time influences their behavior and attitudes. These items are grouped into three dimensions: future (9 items, i.e., “I complete projects on time making steady progress.”), present hedonistic (8 items, i.e., “I do things impulsively”), and present fatalistic (5 items, i.e., “The course of my life is controlled by forces I cannot influence”). The internal consistency, measured by Cronbach’s alpha is 0.77 for the future dimension, 0.45 for the present hedonistic dimension, and 0.68 for the present fatalistic dimension. The observed low alpha value suggests a potential limitation in the reliability of the present hedonistic dimension.

## 5. Statistical Analysis

Statistical preliminary analyses, including reliability analyses and descriptive statistics, were conducted for all the study variables. A factor analysis was performed to identify underlying factors within the Warwick–Edinburgh Mental Well-Being Scale (WEMWBS). Pearson’s correlation coefficient was then executed to examine associations among the variables of interest. Subsequently, multiple regression analyses were conducted to test the impact of self-esteem and time perspectives on mental well-being. The statistical significance level was set at *p* ≤ 0.05. All analyses were conducted using SPSS for statistical support.

## 6. Results

To explore the underlying structure of the data, we performed a factor analysis on the Warwick–Edinburgh Mental Well-Being Scale (WEMWBS) [63] using the principal component method with Varimax rotation. The scale items were loaded onto three primary factors, explaining 65% of the total variance. The factors were labeled and interpreted as follows: energy, positive mood, and sociality. The energy factor encompasses items measuring the individual’s sense of adequately dealing with problems, making appropriate decisions, and exhibiting determination and energy when facing various situations. The positive mood factor includes items reflecting a positive outlook regarding one’s future and a positive perception of a good mood. The third factor, sociality, includes items measuring interest in other people (see Table 1). The Cronbach’s alphas for the three-factor scores were 0.84, 0.69, and 0.76, respectively.

Descriptive statistics were computed for all variables utilized (see Table 2).

Concerning the well-being measure, the Italian 12-item version of WEMWBS displays a total score ranging from 14 to 58, with a mean of 42.06 and a standard deviation of 6.59. The cut-off point was set at 35, meaning that a score below one standard deviation from the mean (about 35.47) was considered indicative of a lower level of mental well-being. In comparison to the standardized Italian sample, incel participants reported relatively low scores for mental well-being (M = 28.22; SD = 7.22).

The Rosenberg Self-Esteem Scale [65] assesses scores ranging from 0 to 30. The cut-off points according to the psychometrics and standardization are as follows: Scores between 16 and 25 fall within the normal range; scores below 16 indicate low self-esteem. As observed in Table 2, incel participants, on average, exhibit generally low self-evaluation (M = 15; SD = 6.70).

Regarding the mean values for the time perspective measured by the Time Perspective Inventory (ZTPI) by D’Alessio et al. [66], we can make some considerations in comparison to the Italian sample used for questionnaire validation. Future perspective: in the Italian sample, the future perspective demonstrates a slightly higher mean (M = 30.79) compared to our incel participants (M = 28.72), suggesting that the incels might be less future-oriented.

Hedonistic present perspective: in the Italian sample, the hedonistic present perspective shows a slightly lower mean (M = 18.61) compared to our incel participants (M = 24.84), indicating that the incel sample might be more focused on the present in pursuing immediate pleasure. 

Fatalistic present perspective: in the Italian sample, the fatalistic present perspective displays a slightly higher mean (M = 19.24) compared to the incel sample (M = 10.80), suggesting that the incel sample may perceive more control over the present compared to the Italian sample.

The Pearson correlation coefficient was calculated to examine bivariate correlations between self-esteem levels, the three dimensions of mental well-being considered, and the three dimensions of the time perspective. As shown in Table 3, the two dimensions of mental well-being, energy, and positive mood positively correlated with self-esteem, the future perspective, hedonistic present, and fatalistic present. Sociality correlates with the hedonistic present but does not correlate with the other dimensions of the time perspective or with self-esteem.

Subsequent multiple regression analyses were conducted to test the influence of self-esteem and time perspectives on the mental well-being factors. The dependent variables in these analyses included energy, positive mood, and sociality, with self-esteem and time perspectives as the independent variables. Table 4a–c show the standardized coefficients of multiple regressions for each dependent variable and the analyses of variance (ANOVAs) associated with the regression model. Regarding energy, the analysis revealed that self-esteem significantly predicts energy levels (Table 4a, Adjusted R2 = 0.69, DW = 1.24). For positive mood, both self-esteem and the hedonistic present perspective have a significant impact (Table 4b, Adjusted R2 = 0.29, DW = 1.91). Regarding sociality, the findings show that the hedonistic current perspective significantly predicts sociality (Table 4c, Adjusted R2 = 0.21, DW = 1.84).

## 7. Discussion

The primary objective of this investigation was to delve into the mental well-being of incels and its associations with self-esteem and temporal perspectives. The examination of the mental well-being of incels revealed a noteworthy trend. Participants exhibited lower levels of mental well-being compared to the standardized Italian sample, indicating a higher prevalence of emotional and social challenges. This aligns with the existing literature suggesting that “romantic loneliness” is linked to lower well-being and negative emotions [46]. These findings underscore the importance of addressing mental well-being issues in this population. Furthermore, our exploration of self-esteem in incels highlighted a prevalent pattern of low self-evaluation. Notably, the role of self-esteem emerged as crucially linked to the energy dimension, one of the three factors of well-being we identified. Hence, low self-esteem in incels exerts a negative influence on the dimension of well-being associated with effectively addressing challenges, making informed decisions, and demonstrating determination and energy in confronting diverse situations. This is in line with previous research identifying the pursuit of self-esteem as a recurring theme in incel forums, with online participation serving as a potential source of self-esteem [9].

The results regarding temporal perspectives reveal an interesting configuration about the time orientations of incels, suggesting reflections on how these individuals conceive and experience time. The data align with the existing literature suggesting that specific dimensions of the temporal perspective are strongly associated with various indicators of mental well-being, predicting up to 40% of their variance [67,68]. Incels appear less future-oriented, focusing their attention on immediate issues and challenges rather than long-term goals. Incel subjects displaying high scores in present hedonistic orientations tend to live in the present, seeking pleasure, novelty, and increasingly gratifying sensations, which leads them to avoid pain. At the same time, the results highlight how incels demonstrate a lower level of fatalistic present perspective compared to the standardized Italian sample, indicating that they perceive greater control over the present. Of note are the data related to the future temporal perspective, which do not show any predictive value on the well-being of incel subjects. Our results are once again aligned with the approach of Zimbardo and Boyd (1999) [57], according to which individuals tend to predominantly assume a time orientation, leading them to focus less on other dimensions. Our subjects highlight high scores on the temporal perspective of the hedonic present and a low score on the future dimension, indicating a lack of evaluation of future consequences, low impulse control, and avoidance-based coping strategies. The propensity of incel individuals exhibiting high scores in present hedonistic orientations to prioritize immediate pleasure and novelty may be rooted in specific psychological mechanisms unique to this group. This inclination could signify an intensified focus on immediate gratification, a preference for momentary pleasures over long-term goals, and potential challenges in impulse control within the incel community. The observed pattern of avoiding pain might reflect a coping strategy where individuals, in their pursuit of pleasure, seek to circumvent discomfort or emotional distress, possibly influenced by the distinct social and psychological dynamics prevalent in incel forums. These behavioral patterns may be indicative of a reduced consideration of future consequences, limited impulse control, and an orientation toward instant emotional satisfaction, underscoring the need for targeted exploration of cognitive and emotional processes within the incel subgroup.

The results of this investigation provide valuable insights into key aspects of the mental well-being of incels, underscoring the need for targeted interventions and practical applications. Consider, for instance, the benefits of enhancing self-esteem in mental well-being interventions. Addressing the mental well-being of this population becomes imperative, especially considering the established link between romantic loneliness, lower well-being, and negative emotions. The prevalent pattern of low self-evaluation among incels highlights the importance of addressing self-esteem in interventions. Strengthening self-esteem, particularly through targeted online platforms where incels often seek validation, could contribute to an overall improvement in the sense of mental well-being.

Moreover, the evident focus on current hedonistic orientations and a reduced emphasis on the future dimension highlight specific areas for intervention. Interventions could involve promoting future-oriented thinking, improving impulse control, and encouraging goal setting to address the observed lack of evaluation of future consequences. In conclusion, our findings could provide crucial insights for designing specific intervention programs aimed at enhancing anticipation, expectation, planning capacity, and future orientation indices in individuals anchored to present hedonism.

## 8. Limitations and Future Directions

The study results must be considered in light of some limitations that warrant discussion. Firstly, the study encountered constraints due to the small sample size and challenges in engaging with the incel community. The sample size was not predetermined through a formal calculation method; instead, exhaustive efforts were made to include as many willing and eligible participants as possible. Nevertheless, it is crucial to acknowledge the significant difficulties faced in engaging with the incel community. A notable strength of this study lies in its direct contact with the incel community.

The use of a non-probability sampling method and reliance on online platforms for recruitment may introduce sampling bias. It is imperative to recognize that incels participating in online forums may not fully represent the entire incel population.

Additionally, respondents voluntarily participated in this study. Therefore, those who participated presented different characteristics than those who chose not to participate. Moreover, one of the limitations of this study is associated with the internal consistency of the hedonistic present dimension.

Future research endeavors should aim for larger sample sizes to validate and extend these findings. A more in-depth engagement with the incel community could offer nuanced insights crucial for understanding fundamental psychosocial aspects. Nevertheless, we assert that this study represents a critical step toward comprehending the psychological dimensions of incels, shedding light on potential avenues for intervention and preventive measures to mitigate the risks associated with the incel ideology.

## 9. Conclusions

In conclusion, our study sheds light on critical aspects of incels’ mental well-being, underscoring the imperative need for targeted interventions. The identified lower mental well-being, coupled with pervasive patterns of low self-esteem, emphasizes the pivotal role of self-worth in this community. Addressing self-esteem emerges as a cornerstone for improving emotional resilience and overall mental health among incels. Recognizing online platforms as potential catalysts for self-esteem enhancement, interventions should prioritize strategies that foster a positive self-evaluation. Our findings highlight self-esteem as a linchpin for navigating challenges, making informed decisions, and exhibiting determination—vital components for fostering a sense of well-being among incels. Consequently, interventions aimed at bolstering self-esteem can serve as transformative pathways toward enhancing the mental health landscape within the incel community.

## Figures and Tables

**Table 1:** Factor structure and factor loadings after VARIMAX of WEMWBS.

Items	Factors		
	Energy	Positive Mood	Sociality
(1) I’ve been feeling optimistic about the future	0.392	0.515	0.184
(2) I’ve been feeling useful	0.509	0.391	0.025
(3) I’ve been feeling relaxed	0.119	0.632	−0.122
(4) I’ve been feeling interested in other people	0.058	0.000	1.004
(5) I’ve had energy to spare	0.799	0.070	0.269
(6) I’ve been dealing with problems well	0.685	0.185	0.168
(7) I’ve been thinking clearly	0.513	0.116	0.063
(8) I’ve been feeling close to other people	0.331	0.110	0.595
(9) I’ve been feeling confident	0.707	0.457	0.113
(10) I’ve been able to make up my own mind about things	0.701	0.201	0.051
(11) I’ve been interested in new things	0.586	0.259	0.160
(12) I’ve been feeling cheerful	0.226	0.793	0.220

**Table 2:** Descriptive statistics, Cronbach’s alpha, and number of valid cases for scales.

Scales	N	M	SD	Min. Value	Max Value	Skewness	Kurtosis	Cronbach’s Alpha
Rosenberg Self-Esteem	58	15.50	6.70	5.00	29.00	0.24	−0.90	0.91
WEMWBS	58	28.24	7.22	12.00	45.00	0.42	−0.15	0.86
Future Perspective	58	28.72	6.33	14.00	44.00	0.20	−0.08	0.77
Hedonistic Present	58	24.84	4.241	16.00	40.00	0.52	1.05	0.45
Fatalistic Present	58	10.80	3.085	5.00	21.00	0.44	1.05	0.68
Number of valid cases (listwise)	58							

**Table 3:** Spearman r correlations between variables.

	Energy	Positive Mood	Sociality	Self-Esteem	Future	Edonistic Present	Fatalistic Present
Energy	1	0.506 **	0.383 **	0.818 **	0.442 **	0.407 **	0.394 **
		<0.001	0.003	<0.001	<0.001	0.002	0.002
Positive Mood	0.506 **	1	0.232	0.508 **	0.351 **	0.404 **	0.288 *
	<0.001		0.080	<0.001	0.007	0.002	0.030
Sociality	0.383 **	0.232	1	0.206	−0.104	0.389 **	0.149
	0.003	0.080		0.121	0.439	0.003	0.269
Self-Esteem	0.818 **	0.508 **	0.206	1	0.484 **	0.334 *	0.447 **
	<0.001	<0.001	0.121		<0.001	0.010	<0.001
Future	0.442 **	0.351 **	−0.104	0.484 **	1	0.048	0.006
	<0.001	0.007	0.439	<0.001		0.720	0.964
Edonistic Present	0.407 **	0.404 **	0.389 **	0.334 *	0.048	1	0.545 **
	0.002	0.002	0.003	0.010	0.720		<0.001
Fatalistic Present	0.394 **	0.288 *	0.149	0.447 **	0.006	0.545 **	1
	0.002	0.030	0.269	<0.001	0.964	<0.001	

* *p* < 0.05; ** *p* < 0.01.

**Table 4:** (**a**) Self-esteem and time perspective predicting energy; (**b**) self-esteem and time perspective predicting positive mood; (**c**) self-esteem and time perspective predicting sociality.

**(a)**								
		**B**	**SE**	**Beta**	**t**	**Sign.**	**Tolerance**	**VIF**
1	(Constant)	−0.364	1.912		−0.190	0.850		
	Self-esteem	0.355	0.049	0.737	7.207	<0.001	0.564	1.774
	Future	0.038	0.046	0.075	0.828	0.412	0.713	1.402
	Edonistic Present	0.133	0.070	0.175	1.894	0.064	0.693	1.443
	Fatalistic Present	−0.032	0.105	−0.031	−0.308	0.759	0.591	1.693
*ANOVA*								
	Model	Sum of Squares	df	Mean Square	F	Sign.		
1	Regression	410.352	4	102.588	29.453	<0.001		
	Residual	181.121	52	3.483				
	Total	591.474	56					
(**b**)								
		B	SE	Beta	t	Sign.	Tolerance	VIF
(Constant)		−0.974	1.971		−0.494	0.623		
Self-esteem		0.113	0.051	0.334	2.228	0.030	0.564	1.774
Future		0.062	0.048	0.173	1.295	0.201	0.713	1.402
Edonistic Present		0.159	0.072	0.297	2.198	0.032	0.693	1.443
Fatalistic Present		−0.018	0.108	−0.025	−0.169	0.867	0.591	1.693
*ANOVA*								
	Model	Sum of Squares	df	Mean Square	F	Sign.		
1	Regression	99.737	4	24.934	6.733	<0.001		
	Residual	192.579	52	3.703				
	Total	292.316	56					
(**c**)								
		B	SE	Beta	t	Sign.	Tolerance	VIF
(Constant)		2.884	1.667		1.730	0.090		
Self-esteem		0.073	0.043	0.279	1.709	0.093	0.564	1.774
Future		−0.073	0.040	−0.262	−1.805	0.077	0.713	1.402
Edonistic Present		0.173	0.061	0.416	2.825	0.007	0.693	1.443
Fatalistic Present		−0.116	0.092	−0.201	−1.260	0.213	0.591	1.693
*ANOVA*								
	Model	Sum of Squares	df	Mean Square	F	Sign.		
1	Regression	38.138	4	9.535	3.598	0.012		
	Residual	137.792	52	2.650				
	Total	175.930	56					

## Data Availability

The data presented in this study are available on request from the corresponding author.
